# A reexamination of information theory-based methods for DNA-binding site identification

**DOI:** 10.1186/1471-2105-10-57

**Published:** 2009-02-11

**Authors:** Ivan Erill, Michael C O'Neill

**Affiliations:** 1 Department of Biological Sciences, University of Maryland Baltimore County, Baltimore, MD, USA

## Abstract

**Background:**

Searching for transcription factor binding sites in genome sequences is still an open problem in bioinformatics. Despite substantial progress, search methods based on information theory remain a standard in the field, even though the full validity of their underlying assumptions has only been tested in artificial settings. Here we use newly available data on transcription factors from different bacterial genomes to make a more thorough assessment of information theory-based search methods.

**Results:**

Our results reveal that conventional benchmarking against artificial sequence data leads frequently to overestimation of search efficiency. In addition, we find that sequence information by itself is often inadequate and therefore must be complemented by other cues, such as curvature, in real genomes. Furthermore, results on skewed genomes show that methods integrating skew information, such as *Relative Entropy*, are not effective because their assumptions may not hold in real genomes. The evidence suggests that binding sites tend to evolve towards genomic skew, rather than against it, and to maintain their information content through increased conservation. Based on these results, we identify several misconceptions on information theory as applied to binding sites, such as negative entropy, and we propose a revised paradigm to explain the observed results.

**Conclusion:**

We conclude that, among information theory-based methods, the most unassuming search methods perform, on average, better than any other alternatives, since heuristic corrections to these methods are prone to fail when working on real data. A reexamination of information content in binding sites reveals that information content is a compound measure of search and binding affinity requirements, a fact that has important repercussions for our understanding of binding site evolution.

## Background

Even though much progress has been made since the first genomic sequences became available, the identification of transcription factor (TF) binding sites in genomic sequences remains a considerable challenge in bioinformatics. In recent years, this problem has been aggravated by the ever-increasing pace of genome sequencing, the realization that junk DNA was a considerable misnomer and by the need to reconcile inferences from high-throughput assays with the underlying genome sequence. New high-throughput technologies, like ChIP-chip and ChIP-Seq [1,2], can contribute significantly to reduce the search space involved in the identification of some TF-binding sites, but theoretical models of binding sites are still required to gain insight into their function and mechanism, and to tackle the general problem of binding site identification in the absence of high-throughput experimental data.

Over the years, the quest for identifying TF-binding sites has taken two natural and complementary approaches, relying either implicitly or explicitly on experimental data. On the one hand, de novo motif discovery methods like MEME, consensus-building, Dyad-Analysis or Gibbs sampling [3-6] use implicit experimental data to uncover overrepresented candidate TF-binding sites in the promoter regions of a set of genes that are known to be co-expressed or co-regulated. On the other hand, different binding site search methods have also been developed to exploit explicit data on the sequence and location of known TF-binding sites [7-10]. In binding site search, data is provided by collections of aligned known sites often referred to as motifs or prototype groups. This work deals with binding site search methods and, in particular, with those relying on the application of information theory to DNA sequences.

### Application of information theory to binding site recognition

Berg & von Hippel introduced a formal approach towards modeling protein-DNA interaction based on the principles of statistical mechanics [10,11]. In their scheme, the contribution to the reduction of binding free energy at each position of a putative binding site is equated with its relatedness to the most representative base occupying that position in the prototype group (i.e. the consensus base), leading to the so called *Heterology Index *(*HI*):

(1) $$HI(l)=\ln\left(\frac{p(S_{l}^{cons})+1/N}{p(S_{l}^{obs})+1/N}\right)$$

where $P(S_{l}^{cons})$ corresponds to the frequency of the consensus base at position *l *of the prototype group, $P(S_{l}^{obs})$ is the frequency of the base observed at position *l *of the site and *N *is the number of sequences in the prototype group (*1/N *acting as a small sample correction to avoid zero frequencies). If one assumes positional independence, a global *HI *for the whole site can be computed by summing *HI(l) *over all site positions [12].

Prior to Berg & von Hippel's statistical mechanics approach, Schneider *et al*. first introduced information theory to the problem of TF-binding site recognition as a robust theoretical framework for defining the interactions between binding sites and their related transcription factors [13]. Based on the theorems of communication over a noisy channel introduced by Shannon [14], information theory can be applied to the recognition of binding sites by transcription factors by acknowledging that recognition of a site by a protein is, essentially, an information process [15,16]. Just as our uncertainty over a message decreases when we receive it, even if it is partly scrambled by noisy interference, the uncertainty about the bases occupying each position of an otherwise unknown sequence decreases once a particular protein does bind it. The amount of uncertainty associated with a variable is called Shannon entropy, typically measured in bits, and can be interpreted as the expectation of its information content:

(2) $$H(X)=-\sum_{i=1}^{N}\left[p\left(X=x_{i}\right)\cdot \log_{2}\left(p\left(X=x_{i}\right)\right)\right]$$

where *N *is the number of possible values (*x*_*i*_) the variable *X *can take.

The expression for Shannon entropy is very similar to the Boltzmann-Gibbs entropy in thermodynamics [17], but they are quite different in substance [18,19]. As expected, Shannon entropy (entropy henceforth) is maximal when all possible states of *X *are equiprobable and independent, since in this situation our uncertainty about which state we will observe is the greatest and thus the amount of information the variable conveys is also maximized. The reduction in uncertainty (or information gain) that takes place during communication over a noisy channel is known as mutual information and is expressed in terms of the difference in uncertainty over the original message (*X*) before and after we receive a version of it (*Y*):

(3) *I*(*X*;*Y*) = *H*(*X*) - *H*(*X*|*Y*)

In the case of binding sites, and again after Schneider *et al*., the a priori uncertainty (*H*_*before*_) over the base occupying position *l *of a single sequence of length *L *is maximal and dictated solely by the background composition of the genome the sequence sits in:

(4) $$H(X)=H_{before}(l)=-\sum_{S\in \Omega }\left[f(S)\cdot (\log_{2}\left(f(S)\right)\right]$$

where *S *corresponds to each of the four possible DNA bases and *f(S) *represents its relative frequency in the genome sequence.

If a particular protein binds a given sequence, however, the amount of uncertainty on the bases at each position of the sequence stems now from the relative frequency of each base at each position of the prototype group for that protein. Thus, the a posteriori entropy (*H*_*after*_) at each position of the sequence becomes:

(5) $$H(X|Y)=H_{after}(l)=-\sum_{S_{l}\in \Omega }\left(p(S_{l})\cdot \log_{2}\left(p(S_{l})\right)\right)$$

where *p(S*_*l*_*) *is the frequency of each base *S*_*l *_at position *l *in the prototype group.

Therefore, for each position, the reduction in uncertainty (or mutual information) experienced when a protein binds to a sequence can be expressed as the difference between a priori (*H*_*before*_) and a posteriori (*H*_*after*_) entropies:

(6) $$I(l)=R_{sequence}(l)=H_{before}(l)-H_{after}(l)=\left[-\sum_{S\in \Omega }\left(f(S)\cdot (\log_{2}\left(f(S)\right)\right)\right]-\left[-\sum_{S_{l}\in \Omega }\left(p(S_{l})\cdot \log_{2}\left(p(S_{l})\right)\right)\right]$$

As defined above, mutual information provides a measure, in bits, of the importance of each position of a binding site in decreasing uncertainty. Assuming positional independence, the term can be summed for all site positions, providing a measure for the whole site.

(7) $$R_{sequence}=\sum_{l=1}^{L}R_{sequence}(l)$$

In the case of a TF-binding site, this equates with the specificity of the site recognition process. By definition, mutual information has a maximum in *H(X)*, corresponding to the case of a noise-free channel (i.e perfect site recognition; *H(X|Y) *= 0), and a minimum in 0 when *X *and *Y *become independent (*H(X|Y) *= *H(X)*). For a given protein, the specificity of the site recognition process is a constant defined by *H(X|Y)*. Therefore, mutual information is maximal whenever *H(X) *is maximized, which in the case of genomic sequences corresponds to an equiprobable base distribution.

In their seminal paper, Schneider *et al*. also introduced a related concept, termed *R*_*frequency*_, to denote the information required to find sites in a genome in terms of both the genome size and the number of sites it contains [13]. The reasoning behind it is quite straightforward. With no additional knowledge, a circular genome of size *G *will contain *G *potential binding sites for a given protein. If we assume that, a priori, all the sites have the same probability (1/*G*) of being bound, we obtain the a priori entropy as:

(8) $$H_{G}=-\sum_{G}\left(\frac{1}{G}\cdot \log_{2}\left(\frac{1}{G}\right)\right)=\log_{2}(G)$$

*H*_*G *_measures the initial uncertainty over any genome position being bound by a single copy of the protein. Then again, if a protein binds *M *specific sites in the genome and we assume that these are bound with equal probability and that the protein does not bind elsewhere, we derive the a posteriori entropy *H*_*M*_:

(9) $$H_{M}=-\sum_{M}\left(\frac{1}{M}\cdot \log_{2}(\frac{1}{M})\right)=\log_{2}(M)$$

Again, we can then express mutual information as the difference between a priori and a posteriori entropies:

(10) $$I=R_{frequency}=H_{G}-H_{M}=\log_{2}(G)-\log_{2}(M)=\log_{2}\left(\frac{G}{M}\right)$$

As defined, *R*_*frequency *_is understood as the amount of information required to distinguish *M *sites from the genomic background. A key observation of Schneider *et al*. was that *R*_*frequency *_approximates *R*_*sequence *_only when considering an equiprobable background. When moving from such an ideal condition, *R*_*sequence *_for a given prototype group decreases steadily because of a net reduction in a priori uncertainty (the restricted background becomes less informative). In contrast, *R*_*frequency *_can stay constant or may increase or decrease heavily as the sites the protein recognizes become, respectively, either scarcer or more abundant in the genome. For a transcriptional regulator, and assuming that function is conserved [20], the number of functional sites (and thus *R*_*frequency*_) will remain constant regardless of the background. For other molecules, such as restriction enzymes, the number of functional sites is effectively the number of binding sites and *R*_*frequency *_will increase or decrease in proportion to their expected frequency in the new background [13]. To circumvent this problem in the second scenario, the authors suggested the use of an ad-hoc modification of *R*_*sequence*_, (R*_sequence_), that approximates *R*_*frequency *_in skewed genomes and equals *R*_*sequence *_in an equiprobable background. This new term turned out to be the Kullback-Leibler divergence or relative entropy [21] and was relabeled accordingly as relative entropy (*RE*) by Stormo [22]:

(11) $$RE(l)=R_{sequence}^{*}(l)=\sum_{S_{l}\in \Omega }\left(p(S^{l})\cdot \log_{2}\left(\frac{p(S_{l})}{f(S_{l})}\right)\right)$$

As in *R*_*sequence*_, positional independence may be assumed in order to generate a global *RE *value for the whole site by summing up *RE(l) *for all positions. The Kullback-Leibler divergence is also measured in bits, allowing direct comparison with *R*_*frequency*_. Following their initial introduction by Schneider *et al*., both *R*_*sequence *_and *RE *have been used by different authors as a measure of the information content in binding motifs [22,23].

Relative entropy was introduced without any formal or intuitive derivation apart from its empirical relationship with *R*_*frequency *_[13]. However, intuitive understanding of *RE *can be easily attained if the term is written in expanded form:

(12) $$RE(l)=\left[-\sum_{S_{l}\in \Omega }\left(p(S_{l})\cdot \log_{2}\left(f(S_{l})\right)\right)\right]-\left[-\sum_{S_{l}\in \Omega }\left(p(S_{l})\cdot \log_{2}\left(p(S_{l})\right)\right)\right]$$

In this new formulation, the second term corresponds to the a posteriori entropy (*H*_*after*_) of *R*_*sequence*_, but the first term represents now the cross-entropy between background and motif frequencies. In essence, cross-entropy measures the amount of information required to express the observed motif frequencies in terms of their genomic counterparts. More intuitively, by simple manipulation of the *RE *formula:

(13) $$RE(l)=\left[-\sum_{S_{l}\in \Omega }\left(\frac{p(S_{l})}{f(S_{l})}\cdot f(S_{l})\cdot \log_{2}\left(f(S_{l})\right)\right)\right]-\left[-\sum_{S_{l}\in \Omega }\left(p(S_{l})\cdot \log_{2}\left(p(S_{l})\right)\right)\right]$$

cross-entropy can be conceptualized as a weighted version of a priori entropy (*H*_*before*_). For each of the four possible bases in a motif position (*S*_*l*_), a priory entropy is now weighted up or down depending on the ratio between the motif and background frequencies for that particular base *p(S*_*l*_*)*/*f(S*_*l*_*)*. In this manner, if a base is for instance underrepresented in the genome but conserved in the motif, its contribution to the a priori entropy will become higher and, consequently, *RE(l) *will increase. Conversely, a conserved base that is overrepresented in the genome will contribute less. As a consequence, in a skewed background *RE *is larger for motifs relying on underrepresented bases, agreeing with *R*_*frequency *_predictions, in which "rarer" sites require additional information in order to be found.

### Information theory-based methods for TF-binding site search

Apart from the aforementioned *Heterology Index *of Berg & von Hippel, which serves as a search function directly, several other methods have been proposed over the years to search for TF-binding sites based on the availability of a prototype group of experimentally validated binding sites. Even though some of them were proposed before the introduction of the information theory framework, they all can be derived from the expressions for *R*_*sequence *_and *RE *seen above.

Staden first proposed a simple yet powerful index to evaluate the likelihood that a sequence was a binding site for a given protein [24]. This method was later refined by Schneider [23], who showed that it could be derived formally from the expression of *R*_*sequence *_and labeled it *R*_*i*_, as the information content of an individual binding sequence *i*:

(14) $$R_{i}(l)=\left[-\sum_{S\in \Omega }\left[f(S)\cdot \log_{2}\left(f(S)\right)\right]\right]-\left[-\log_{2}\left(p(S_{i,l})\right)\right]=H_{before}-\left[-\log_{2}\left(p(S_{i,l})\right)\right]$$

where *p(S*_*i*, *l*_*) *is the frequency of occurrence in the prototype group of the base *S *observed at position *l *of the query sequence *i*. As in the case of *R*_*sequence*_, positional independence is assumed and the score for the full sequence *i *is the sum of *R*_*i*_*(l) *over all its positions.

Later on, Hertz *et al*. proposed the use of a term deriving from *RE *to search for putative binding sites [25,26]:

(15) $$I_{i}^{seq}(l)=p(S_{i,l})\cdot \log_{2}\left(\frac{p(S_{i,l})}{f(S_{i,l})}\right)$$

that explicitly takes into account the background genomic frequencies *f(S*_*i*, *l*_*) *and that again assumes positional independence to obtain an additive score for the full site. In this work we label this term *I*_*seq *_to avoid confusion with the relative entropy (*RE*) term from which it derives.

A fundamental problem of both *I*_*seq *_and *R*_*i *_is that they discard information on the relative importance of each position within the motif. This is clearly illustrated by a simple example. Suppose that for a given position *a *of a motif we have prototype frequencies p_a_(A) = 0.6, p_a_(C) = 0.4, p_a_(T) = 0.0 and p_a_(G) = 0.0. If we observe a C in our query sequence, then *R*_*i*_*(a) *= *H*_*before*_-log_2_(0.4). It is easy to see, however, that if position *b *of the motif has prototype frequencies p_b_(B) = 0.2, p_b_(C) = 0.4, p_b_(T) = 0.2 and p_b_(G) = 0.2 and we again observe a C in the query sequence, *R*_*i*_*(b) *= *H*_*before*_-log_2_(0.4). That is, *R*_*i *_is assigning the same score to a C observed in a relatively well conserved position (*a*) and to a C observed in a nearly random one (*b*). This result is counterintuitive in the sense that we would expect that a match in a conserved position be more significant than a match in a poorly conserved one. O'Neill pointed out this problem and suggested two alternative methods to take into account the importance, or weight, of each position in the prototype group [27,28].

A first obvious means to circumvent the loss of information about the importance of each position within the motif is to enter it explicitly into the search function as a weighting factor [27]. O'Neill applied this weighting approach on the *Heterology Index *(*HI*) of Berg & von Hippel, even though the principle can be applied as well to all the search functions described above:

(16) $$R_{sequence}\cdot BvH=\sum_{l=1}^{L}R_{sequence}(l)\cdot HI(l)$$

A more elegant solution to the same problem involves the use of a differential *R*_*sequence *_term. In this approach, *R*_*sequence*_*(l) *is calculated both before (^-^) and after (^+^) the addition of the query sequence to the prototype group [28]. It follows that if the query sequence concurs with the prototype, the expression:

(17) $$R_{sequence}^{'}(l)=R_{sequence}^{-}(l)\cdot \left(R_{sequence}^{+}(l)-R_{sequence}^{-}(l)\right)$$

will yield a positive value because *R*^+^_*sequence*_*(l) *will be improved by the addition, whereas a query sequence discordant with the prototype will result in a negative value.

Historically, there has been substantial dissention among the appropriate definition of information content (*R*_*sequence *_or *RE*) [20,22,23], the suitability of the positional independence assumption [29,30] and the relative efficiency of the abovementioned methods and later variants [9,22,28] for locating TF-binding sites in both equiprobable and skewed genomic backgrounds. Unfortunately, at the time most of these methods were developed there was not enough experimental data to test their shortcomings and advantages in a real biological setting and, even in relatively recent studies, most search efficiency results have been presented on randomly generated backgrounds [9]. In this work we make use of newly available data on experimentally validated binding sites across different species to assess the limits of the different search methods, to gauge the suitability of alternative definitions of information content and to expose the drawbacks of benchmarking on random sequence. The results reported here point at substantial misconceptions in the derivation of information theory methods, leading us to propose a complete reformulation of the concept of information content in binding sites. Consequently, they have deep implications for the understanding of binding site evolution and for the assessment of binding site sequence function in the search and recognition processes.

## Results and discussion

### Assessment of search efficiency in an equiprobable genomic background

**Figure 1:**
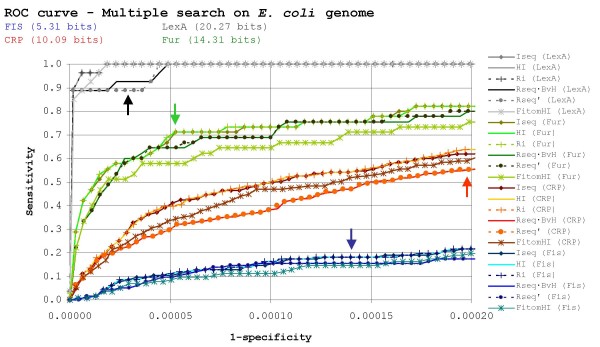
**Search efficiency in the *E. coli *genome**. ROC curves for different IT-based binding site search methods attempting to locate known LexA, Fur, CRP and Fis sites on the *E. coli *genome. The plot is scaled to encompass a 1/10 true to false positive ratio for the transcription factor with the largest number of known sites (CRP; 210 sites). Vertical arrows indicate this same ratio for all transcription factors.

To assess the efficiency of the different information theory-based methods on the problem of locating TF-binding sites on an equiprobable genomic sequence, searches for four different transcription factor binding sites were conducted against the *Escherichia coli *genome (50.8% GC) using collections of known binding sites derived from the literature. The results shown in Figure 1 correspond to Receiver-Operating Characteristic (ROC) curves [31] for all methods when attempting to locate binding sites of four different transcription factors (FIS, CRP, Fur and LexA) in the *E. coli *genome. To generate the ROC curves for each transcription factor, all its experimentally validated sites present in the genome were considered positives, while all other possible genome positions were considered negatives. This is necessarily a strong assumption (as some false positives might indeed be non-experimentally validated true sites), but the same assumption holds for all the assessed methods. As expected, all search methods perform better for transcription factors with more conserved motifs (i.e. larger *R*_*sequence*_). However, Figure 1 also reveals remarkable differences and similarities that had not previously been assessed.

The fact that *I*_*seq *_and *R*_*i *_perform similarly has been already pointed out [22] and should not be surprising, since the base distribution in *E. coli *is almost equiprobable and the methods derive, respectively, from *R*_*sequence *_and *RE*, which are known to be equal on equiprobable backgrounds [13]. Likewise, the similar results of *R*_*sequence *_· *BvH *and *R*^'^_*sequence *_had been noted previously [28]. At first glance, though, a more intriguing result stems from the nearly exact equivalence of Berg & von Hippel (*HI*) and *R*_*i *_indices, since they derive from conceptually different theoretical frames. Careful examination of the Berg & von Hippel index, however, reveals that it does not fulfill the role for which it was intended. In principle, *HI *ought to take into account the fitness of each query site position by contrasting it with the consensus base at that same position in the prototype group. However, a simple manipulation of the original *HI *formulation reveals that it performs virtually the same computation carried out by *R*_*i*_. Specifically, the expression for *HI *at each site position can be rewritten as:

(18) $$HI(l)=\ln\left(\frac{p(S_{l}^{cons})+1/N}{p(S_{l}^{obs})+1/N}\right)=\ln\left(p(S_{l}^{cons})+1/N\right)-\ln\left(p(S_{l}^{obs})+1/N\right)$$

and since the prototype group does not change for different query sites, the first term of the expression is effectively a constant (as is *H*_*before *_in the expression for *R*_*i*_). Therefore, when summed up for all site positions, *HI *can be written as:

(19) $$HI=(const.)-\sum_{l=1}^{L}\left[\ln\left(p(S_{l}^{obs})+1/N\right)\right]$$

which is, for the intents and purposes of a binding-site search function, equivalent to:

(20) $$R_{i}=(const.)+\sum_{l=1}^{L}\left[\log_{2}\left(p(S_{i,l})\right)\right]$$

A new index, here termed *FitomHI*, that does explicitly take into account the difference between consensus and observed bases is introduced below, and its results are also plotted in Figure 1:

(21) $$FitomHI(l)=\frac{p(S_{l}^{cons})}{p(S_{l}^{obs})}\cdot \log_{2}\left(p(S_{l}^{obs})\right)$$

By using the ratio between consensus and observed frequencies as a multiplicative factor on a stripped-down version of *R*_*i*_, *FitomHI *ensures that the intuitive relationship derived by Berg & von Hippel is explicitly taken into account when scoring candidate sites. As it can be seen in Figure 1, however, the *FitomHI *index does not outperform other methods (such as *R*_*i*_) suggesting that the hypothesis behind the Berg & von Hippel scheme might have been misguided.

The relatively poor performance of the *FitomHI *index points up another obvious but nonetheless important observation regarding the results of Figure 1. As it can be readily seen, methods that do not take into account the importance of each position in the prototype group (i.e. non-weighted methods: *R*_*i*_, *HI*, *I*_*seq*_) consistently outperform those that do integrate this factor (weighted methods: *R*_*sequence *_· *BvH*, *R*^'^_*sequence*_), with the proposed *FitomHI *index falling somewhat in between. As in the case of *FitomHI*, this is at first glance an unexpected and counterintuitive result, since weighted methods have been shown previously to perform well in searching [9] and to excel at ranking TF-binding sites according to their experimental binding affinity [27]. Moreover, both the notion of positional weighting and of a ratio between consensus and observed bases are intuitively appealing [28].

The reason why weighted methods perform poorly in search mode when compared to non-weighted ones is, nonetheless, relatively straightforward. By down-weighting poorly conserved positions, weighted methods concentrate their scoring on a smaller number of conserved positions, thereby increasing the chances that "correct" bases might appear by chance at those positions during a genome-wide search and thus leading to a larger number of false positives. Conversely, non-weighted methods bestow the same importance to all motif positions, lowering the odds that false positives may arise by chance. In this context, *FitomHI *can be seen as a crude weighted method, since it is taking into consideration part of the information profile through its explicit use of the consensus-to-observed frequency ratio.

The superiority of non-weighted methods over weighted ones in binding site searches raises important questions regarding site recognition by proteins. To a certain extent, the problem of ranking binding sites can be equated with binding affinity, while the search problem ostensibly equates with the protein's ability to locate its binding sites. Traditionally, it has been assumed that binding site affinity and binding site location are intrinsically linked at the protein level and, thus, models developed for one problem have applied to the other without much consideration. However, the disparity in performance between weighted and non-weighted methods on the search problem suggests that this may not be a good practice. The intuitive concepts behind weighted methods and the Berg & von Hippel approach were initially introduced to deal with the ranking problem and thus they may not apply as well to the related search problem. Furthermore, the main difference between both kinds of methods (i.e. positional weighting) points to a mechanistic difference between these two different modes of action of DNA-binding proteins.

The fact that non-weighted methods outperform weighted ones in genome-wide searches suggests that information lying in poorly conserved motif positions is being used actively by the protein to discern true binding sites against the genomic background. As mentioned above, the equal appraising of all site positions by non-weighted methods has the net effect of reducing the number of possible false positives. However, given the nature of protein-DNA interactions, it is unlikely that such discrimination is achieved by specific recognition on all motif positions. Instead, the uniform use of all site positions in non-weighted methods seems to be taking into account secondary information (e.g. AT-richness) residing in poorly conserved positions that can be of relevance to the protein in order to make non-specific contacts or as a requirement for optimal curvature or bendability. In contrast, the better performance of weighted methods in ranking sites according to their binding affinity indicates that conserved motif positions are the main players in determining the strength of a site [27]. In agreement with this, the mean difference in search efficiency between weighted and non-weighted methods decreases (from 15.1% for Fis to 0.3% for LexA) as motif conservation increases, suggesting that there is an increasing dependence on secondary information sources for proteins targeting less conserved sites, as would be expected in that these sites remain functional.

The resulting disparity between weighted and non-weighted methods is not the only clue pointing towards the use of additional information in the process of site location. At *R*_*sequence *_= 10.09 bits, CRP is substantially underspecified to cover its 210 experimentally validated sites, since *R*_*frequency *_predicts that at least 14 bits should be necessary to specifically locate 210 sites on the *E. coli *genome. This implies that, on average, 28% of the information required to specify true CRP sites is not present as positional information in *R*_*sequence*_. In fact, the estimated number of sites for CRP based on the equivalence between *R*_*sequence *_and *R*_*frequency *_is about 4,300, but even on a 1/30 true- to false-positive ratio (i.e. accepting ~6,100 false positives) the best search method is only able to retrieve 80.7% of the true sites (data not shown). This means that nearly 20% of true CRP sites are left unaccounted for when using information theory-based methods for locating them. Moreover, the set of non-located true sites displays very low *R*_*sequence *_(6.17 bits), suggesting again that other sources of information should be exploited to improve these predictions [32]; the protein could not function were it actually faced with the challenge of 100,000 pseudo-sites as this low information level suggests. Experimental results have already hinted at the existence of several complementary sources of information for site location, such as curvature [33-36], pre-recruitment or cooperative binding [37-39]. As formulated originally, information theory-based methods cannot take into account this additional information, but they provide a robust theoretical foundation to develop more complex methods that incorporate it explicitly. In fact, several higher order models based on information theory that include contextual information have already been proposed [40-42].

### Assessment of search efficiency on skewed artificial backgrounds

As mentioned above, skewed backgrounds disrupt the equivalence between *R*_*sequence *_and *R*_*frequency*_, as the decrease in background entropy (*H*_*before*_) reduces the net amount of mutual positional information (*R*_*sequence*_) while, depending on their composition, sites can become either more or less frequent (*R*_*frequency*_) in the skewed background. To correct for this effect, Schneider *et al*. introduced the concept of *Relative Entropy *(*RE*), from which the search method *I*_*seq *_derives. By taking explicitly into account the background frequency of the bases observed in a site, both *RE *and *I*_*seq *_compensate for the scarcity or overabundance of each particular base in the genome. To make a rigorous assessment of the differences between weighted and non-weighted methods on skewed backgrounds, here we introduce two new search methods based on the weighted scheme proposed by O'Neill [28]. Essentially, both methods are modifications of those proposed previously (*R*_*sequence *_· *BvH *and *R*^'^_*sequence*_), but using *RE *instead of *R*_*sequence *_as the weighting factor:

(22) $$RE\cdot BvH=\sum_{l=1}^{L}RE(l)\cdot HI(l)$$

and

(23) *RE*' (*l*) = *RE*^- ^(*l*) · (*RE*^+ ^(*l*) - *RE*^- ^(*l*))

**Figure 2:**
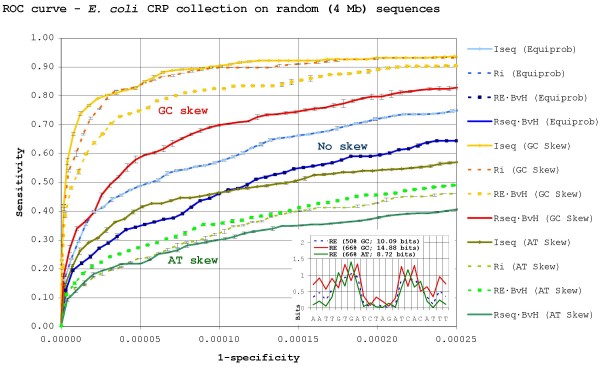
**Search efficiency for *E. coli *CRP sites in a skewed random background**. ROC curves for search methods trying to locate 210 CRP binding sites on randomly generated backgrounds. The ROC curve depicts the mean and standard deviation of three independent experiments (searches against three independently genrerated backgrounds). The plot is scaled to encompass a 1/10 true to false positive ratio (2100 false positives) in the equiprobable background. *RE' *results, which completely overlap *RE · BvH *ones, are not shown for clarity. The *RE *profiles for CRP against the different backgrounds are shown in the bottom-right inset.

**Figure 3:**
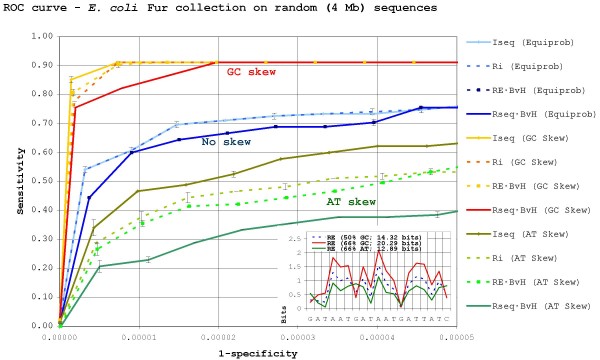
**Search efficiency for *E. coli *Fur sites in a skewed random background**. ROC curves for search methods trying to locate 45 Fur binding sites on randomly generated backgrounds. The ROC curve depicts the mean and standard deviation of three independent experiments (searches against three independently genrerated backgrounds). The plot is scaled to encompass a 1/10 true to false positive ratio (450 false positives) in the equiprobable background. *RE' *results, which completely overlap *RE · BvH *ones, are not shown for clarity. The *RE *profiles for Fur against the different backgrounds are shown in the bottom-right inset.

Figure 2 and Figure 3 show the ROC curves for information theory-based methods attempting to locate, respectively, CRP and Fur binding sites against equiprobable, 66% GC- and 66% AT-skewed randomly generated backgrounds, with their *RE *profile plots shown as insets in the bottom-right corner. The curves show the mean and standard deviation of three independent experiments and thus reveal that the differences between the observed methods are statistically significant. As it can be seen, all methods substantially improve their results on equiprobable random backgrounds when compared to those obtained on the *E. coli *genome. Even though the *E. coli *genome is nearly equiprobable, this is to be expected, since naive random sequences are not very good approximations of genome sequences, in which certain word frequencies can be heavily biased [43,44] despite the overall base frequencies. As a consequence, reports on the effectiveness on TF-binding site search methods based on searches against random sequences should be approached with some caution.

The motifs for both CRP and Fur TF-binding sites are manifestly AT-rich and, as expected, binding sites for both proteins become more or less apparent in, respectively, GC- or AT-skewed backgrounds. In accordance with this fact, *RE*-based methods (i.e. *I*_*seq *_and *RE · BvH*), which have been devised to take into account explicitly the deviation of sites from the background skew, consistently outperform *R*_*sequence*_-based methods on skewed backgrounds, although there are noticeable differences depending on the background skew and the motif searched. In GC-rich backgrounds, both AT-rich sites are relatively easy to locate. Thus, the downplaying of the few G/C positions carried out by the *RE *non-weighted method (*I*_*seq*_) is not a strong advantage over its *R*_*sequence *_counterpart (*R*_*i*_). This does not hold true for weighted methods, which discard a large proportion of the AT-rich sites by focusing on conserved positions, allowing *RE · BvH *to clearly outperform *R*_*sequence *_· *BvH *when looking for CRP. On the other hand, searches on AT-rich backgrounds yield a completely different picture. By playing down the dominant A/T positions in the motifs and emphasizing the scant G/C ones, *RE *substantially alters the shape of the information profile. As a consequence, *RE*-based methods are able to separate Fur and CRP sites from the AT-rich background much more efficiently than *R*_*sequence*_-based methods, and this applies both to weighted and non-weighted methods.

### Assessment of search efficiency on skewed genomes

**Figure 4:**
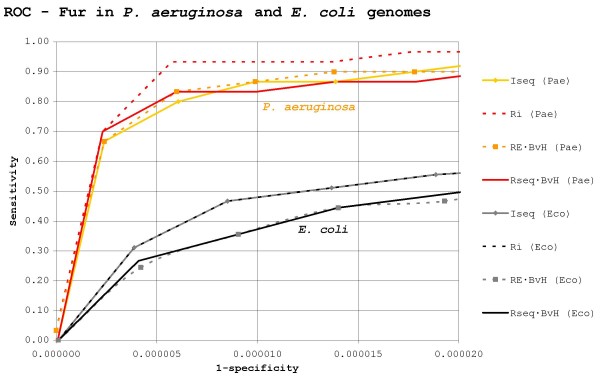
**Search efficiency for Fur sites in *E. coli *and *P. aeruginosa***. ROC curves for search methods trying to locate *P. aeruginosa *and *E. coli *Fur binding sites on, respectively, *P. aeruginosa *and *E. coli *genomes. Abbreviations: Eco – *E. coli*, Hin – *H. influenzae*. The plot is scaled to encompass a 1/10 true to false positive ratio (320 false positives) in *P. aeruginosa*.

**Figure 5:**
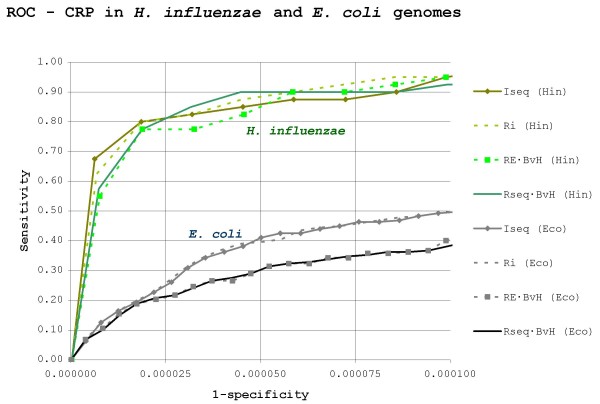
**Search efficiency for CRP sites in *E. coli *and *H. influenzae***. ROC curves for search methods trying to locate *H. influenzae *and *E. coli *CRP binding sites on, respectively, *H. influenzae *and *E. coli *genomes. Abbreviations: Eco – *E. coli*, Hin – *H. influenzae*. The plot is scaled to encompass a 1/10 true to false positive ratio (450 false positives) in *H. influenzae*.

The results of search methods on randomly generated skewed backgrounds support the notion that deviation from the background skew is an important element for proteins targeting binding sites in skewed genomes. Accordingly, it has been suggested that the use of *RE*-based methods is indicated when looking for TF-binding sites in skewed genomes [13,22,25]. However, one must remember that these results were based on artificial sequences. Exploiting the recent availability of data on both CRP and Fur regulons in species with AT- and GC-skewed genomes (*Pseudomonas aeruginosa *and *Haemophilus influenzae*), searches for CRP and Fur binding sites against real genomic backgrounds were carried out to test the validity of this hypothesis. ROC curves for *RE*-based and *R*_*sequence*_-based methods trying to locate *P. aeruginosa*, *H. influenzae *and *E. coli *Fur and CRP binding sites in their corresponding genome sequences are displayed in Figure 4 and Figure 5.

**Figure 6:**
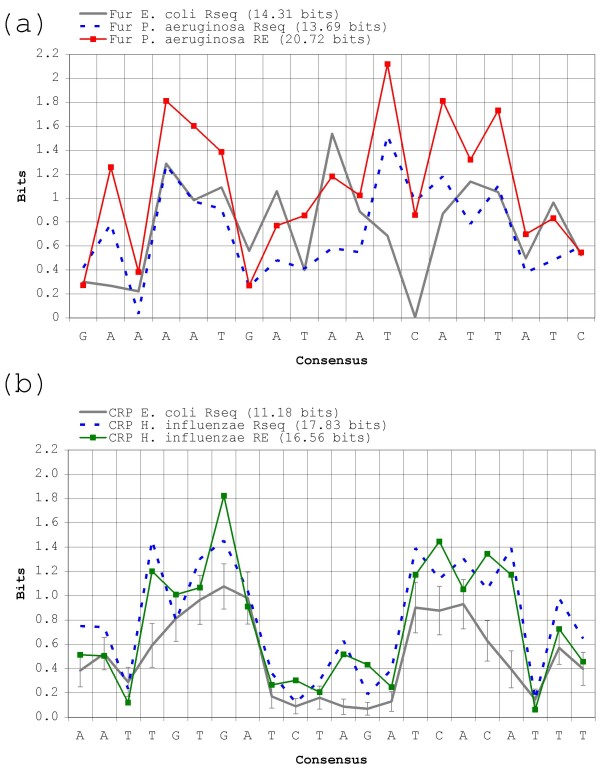
**Information profile for *P. aeruginosa *Fur and *H. influenzae *CRP motifs**. (A) *R*_*sequence *_and *RE *profiles for Fur on the *P. aeruginosa *genome. (B) *R*_*sequence *_and *RE *profiles for CRP on the *H. influenzae *genome, and for the mean *R*_*sequence *_profile obtained from 10,000 45-site subsamples of the 210 *E. coli *binding sites. Vertical bars show the standard deviation.

A main result from the above searches against real genomic backgrounds is that *RE*-based methods tend to perform worse than, or at best similarly to, *R*_*sequence*_-based ones, in contrast to the results obtained previously on randomly generated backgrounds (Figure 3). This is particularly true for Fur in *P. aeruginosa *(Figure 4). In this setting, the *RE*-derived method *I*_*seq *_performs on a par with the weighted *R*_*sequence *_· *BvH *index, while its *R*_*sequence*_-based equivalent (*R*_*i*_) produces the best result. The *R*_*sequence *_and *RE *profiles for the *P. aeruginosa *Fur prototype group are shown in Figure 6a. As it can be seen, the *P. aeruginosa *Fur profile shape is different from that observed in *E. coli*, but its consensus sequence and overall *R*_*sequence *_remain highly similar. Moreover, searches for *P. aeruginosa *Fur sites using the *E. coli *prototype group make the difference between *R*_*i *_and *I*_*seq *_even starker (data not shown). Therefore, the poor efficiency of *I*_*seq *_in this setting cannot lie in a dramatic change of the prototype group, but specifically in the transition from a randomly generated background to a true genome.

**Figure 7:**
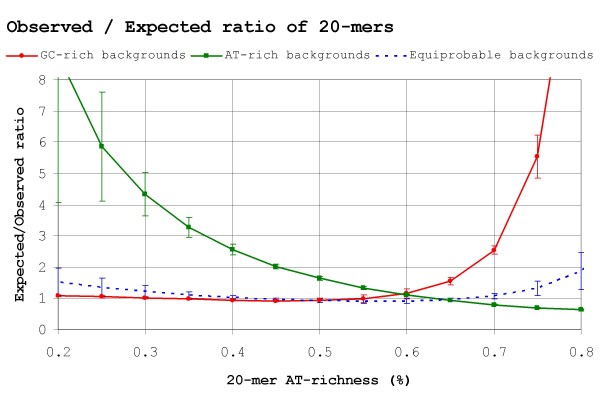
**Observed vs. expected frequency of 20-mers in genomes**. Mean ratio between observed and expected 20-mers in real genomes versus randomly generated sequences. Ratios were computed independently for 3 different genomes and 3 random sequences of similar %GC composition. Vertical bars show the standard deviation of these ratios. Genomes used for calculations: *E. coli *str. K-12 substr. MG1655 [50.8% GC], *P. aeruginosa *PAO1 [66.6% GC], *H. influenzae *Rd KW20 [38.1% GC], *Colwellia psychrerythraea *34H [38.0% GC], *Salinibacter ruber *DSM 13855 [66.2% GC], *Thiobacillus denitrificans *ATCC 25259 [66.1% GC], *Enterococcus faecalis *V583 [37.5% GC], *Anaplasma marginale *str. St. Maries [49.8% GC] and *Nitrosococcus oceani *ATCC 19707 [50.3% GC].

It is a counter-intuitive fact that in *P. aeruginosa *and other genomes with similar GC-skew the distribution of continuous AT-rich stretches is markedly non-uniform when compared to that of randomly generated GC-skewed backgrounds (Figure 7). Remarkably, the corresponding effect is observed in *H. influenzae *and other AT-rich genomes, which show both a higher number of GC-rich stretches and a lower number of AT-rich stretches than expected. The net result of these deviations from expectation is that the overweighting of anti-skew (and underweighting of pro-skew) positions carried out by *RE*-based methods backfires when looking for sites in real skewed genomes. This mismatch is most obvious when looking for Fur sites (70.72% AT) in the GC-rich *P. aeruginosa *genome (Figure 4), where the distribution of 70% AT stretches more than doubles the random expectation (Figure 7). This leads *RE*-based methods to yield high false positive rates because many AT-rich stretches with other functions do easily qualify as putative Fur sites when examined under *RE*. This constitutes a solid blow to *RE*-based methods, because *P. aeruginosa *Fur sites are precisely the type of problem *RE *was introduced to deal with [45]. In the case of *H. influenzae *CRP sites (69.89% AT), the mismatch is not so large, because 70% AT stretches do not deviate so strongly from expectation (down by 25%). However, it is still enough to render the weighting scheme of *RE *useless, if not counterproductive (Figure 5).

As in the case of equiprobable backgrounds, these results stress again the need to validate search methods against real genomic data in order to derive meaningful results. Moreover, they also point out that the rationale for the derivation of *RE *and its resulting indices (*I*_*seq*_, *RE · BvH*) may be partly flawed. Schneider *et al*. proposed *RE *as a way to extend the equivalence between *R*_*sequence *_and *R*_*frequency *_in equiprobable backgrounds to skewed genomes. This line of reasoning has later been utilized by Stormo and coworkers [25,26]. A main flaw in their argument stems from the fact that *R*_*frequency *_was derived from the expected frequency of occurrence of sites in a uniform background. As noted above, however, in a real skewed genome the occurrence of anti-skew stretches can be far from uniform, and the net effect of this biased distribution is to make the use of *RE *meaningless or even counterproductive, as in the case of Fur sites in *P. aeruginosa*.

*R*_*sequence *_is free from the artifacts created by deviations in oligonucleotide distribution and similar factors involved in the search problem in that it is a measure only of positional information. As such, it is a more reliable indicator of motif positional information content than *RE*. Therefore, *R*_*sequence*_-derived methods (e.g. *R*_*i*_) should be expected, on average, to perform better than *RE*-based ones. It should be pointed out here that an often implied argument for the use of *RE *over *R*_*sequence*_, the advent of negative information content in skewed genomes, is based on a misconception. Computing *R*_*sequence *_for a collection of *E. coli *sites against a skewed background may indeed generate negative *R*_*sequence *_values, but this perplexing result is an artifact of the transplantation of the *E. coli *motif onto a skewed background rather than a fault in the formulation of *R*_*sequence*_. On a skewed genome, the a priori entropy (*H*_*before*_) is reduced because of the background skew. To obtain negative values for *R*_*sequence*_, nucleotide frequencies in some positions of the binding motif must be close to equiprobability. In this case, the a posteriori entropy (*H*_*after*_) will be greater than the a priori one, leading to a negative *R*_*sequence *_value. This is indeed the case of most non-conserved positions in many *E. coli *motifs when evaluated on a skewed background. However, it is easy to see that, for real binding sites evolving in a skewed genome, positions that are not important for binding will remain at background genomic frequencies (instead of being actively selected towards equiprobability), thus leading to positive or, at the most, zero values for *R*_*sequence*_.

The failure of *RE*-based methods to outperform *R*_*sequence*_-based ones in real genomes casts serious doubts on the validity of this approach and its main underlying assumption, the equivalence between *R*_*frequency *_and *R*_*sequence*_. However, the inadequacy of *RE *to deal with non-uniform n-mer frequencies is not the only result pointing to a demise of the equality between *R*_*frequency *_and *R*_*sequence*_. A main corollary of the hypothesis for deriving *RE *is the assumption that when a genome drifts towards skew in its base composition, DNA-binding proteins shall evolve to recognize binding sites with an anti-skew composition, thus maximizing the efficiency of binding site location at a lesser cost in overall base conservation. This line of reasoning was explicitly developed by Schneider *et al*. They noted that sites with anti-skew composition would eventually lose positional information (*R*_*sequence*_) in the course of evolution, since selective pressure towards site conservation would be reduced because the site would be over-specified and therefore easier to locate [13]. In other words, integrating anti-skew composition in a measure of positional information, as *RE *does, would compensate for the loss of standard positional information (*R*_*sequence*_). The results shown in Figure 4 and Figure 5, however, suggest that this is not generally the case.

On the one hand, the *P. aeruginosa *Fur protein seems to control a regulon of about the same size (32 known sites) as that of *E. coli *Fur (51 known sites) and its motif positional information content (*R*_*sequence *_= 13.69 bits) is similar to that of *E. coli *Fur (*R*_*sequence *_= 14.31 bits). The ratio between these *R*_*sequence *_values is in accordance with previous estimates for Fur information content based on optimized alignments for a smaller number of sites (20 sites and 18.6 bits for *P. aeruginosa *Fur; 24 sites and 19.6 bits for *E. coli *Fur [46]). Sitting in the 66.56% GC *P. aeruginosa *genome, however, the Fur motif has a markedly anti-skew composition (70.72% AT). The resulting *RE *value of 20.72 bits should allow Fur to target specifically as few as two sites in the whole genome. Thus, if the main hypothesis behind *RE *were true, *P. aeruginosa *Fur could have discarded a substantial part of its positional information (*R*_*sequence*_) by relying on anti-skew composition. Instead, *P. aeruginosa *Fur maintains a *R*_*sequence *_value similar to that of *E. coli *Fur despite the loss in genomic entropy (*H*_*before*_) due to genomic skew (i.e. *P. aeruginosa *Fur sites are more conserved that *E. coli *Fur sites). On the other hand, in the 38.1% GC-rich *H. influenzae *CRP sites are strongly conserved (*R*_*sequence *_= 17.83 bits) in comparison to those of *E. coli *CRP (10.09 bits). A plausible explanation for this effect could be an error due to small sample (there are 45 described CRP sites in *H. influenzae *for 210 in *E. coli*), but using the small sample correction proposed by Schneider *et al*. on H. influenzae CRP sites does only decrease *H. influenzae *CRP *R*_*sequence *_to 16.51 bits [13]. Moreover, iterated sub-sampling of the 210 *E. coli *sites into 45-site prototype groups does not yield enough deviation to explain the observed 7 bit increase either (Figure 6b). In fact, the maximum *R*_*sequence *_value for any of the 10,000 sampled groups is still 4 bits away (13.89 bits) from the *H. influenzae *profile. As mentioned above, a corollary of *RE *is the prediction that DNA-binding motifs should tend to evolve against the skew in order to profit from easier location. However, the *H. influenzae *CRP protein is not relying substantially on anti-skew composition to detect its sites. Instead, efficient location of these AT-rich sites in the AT-rich background of *H. influenzae *seems to be based entirely on increased *R*_*sequence*_. If anything, both *H. influenzae *CRP and *P. aeruginosa *Fur sites seem to have adapted towards the skew, not against it. The *P. aeruginosa *Fur motif is 70.72% AT (for 74.71% AT in *E. coli*), while the *H. influenzae *CRP motif is 69.89% AT (for 64.68% AT in *E. coli*). In summary, both motifs have moved towards the skew, and both have become more conserved.

### A reappraisal of information content in binding sites

Since its introduction in 1986 [13], the assumption of equality between *R*_*frequency *_and *R*_*sequence *_that lies at the core of *RE *has been considered a de facto axiom of information theory applied to binding sites and has shaped the way we think about binding site search, specificity and evolution. However, the results presented above stand in open contradiction with the predictions made by this hypothesis and thus beg us to reconsider its validity and applicability.

In 2000, Schneider showed by means of a genetic algorithm that, given some constraints, *R*_*sequence *_would evolve towards *R*_*frequency *_[20]. Later on, Kim *et al*. applied a more formal mathematical treatment to the same problem and concluded that deviations between *R*_*sequence *_and *R*_*frequency *_are constrained to a very small range [47]. An important assumption in both analyses is the use of an on-off switch model for the transcription factor (i.e. sites are either recognized or not according to a threshold). Even though some studies suggest that the transition from sites to non-sites is relatively sharp for some transcription factors [36], the use of an on-off model is still a strong assumption, since it is well known that transcription factors present a varied range of binding affinities for the binding sites they recognize [48-51]. Therefore, the use of a "black/white" approach centers the ensuing analysis exclusively on the problem of how the protein identifies its target sites in the genome, disregarding completely any functional requirements of the protein for differentially regulating its different binding sites. It is worth noting here that a last implicit constraint in both analyses is the assumption of an equiprobable background. This is important because, as it will be shown, it is only in such a context that *R*_*sequence *_equates to a significant degree with search and, therefore, with *R*_*frequency*_.

As outlined in the introduction, *R*_*sequence *_and *R*_*frequency *_measure subtly different things. *R*_*sequence *_is associated with the uncertainty of the recognition process, while *R*_*frequency *_measures the uncertainty in terms of distinguishing a sequence from the genomic background. Therefore, *R*_*frequency *_is intrinsically linked to the search problem, but *R*_*sequence *_is only partly related to it. In an equiprobable background, where the equality between *R*_*sequence *_and *R*_*frequency *_was first postulated [13], *R*_*sequence *_is substantially related to the search problem. This is because location of binding sites by the protein proceeds by Brownian diffusion and contacts with DNA in a random manner. Contacts between DNA and protein can be non-specific (totally electrostatic) with non-sites or specific for sites according to the protein profile (true sites and pseudo-sites) [52]. Thus, search efficiency improves as the affinity of the protein for its true sites increases (i.e. *R*_*sequence *_increases), since this implies that fewer genomic positions will qualify as pseudo-sites for the protein. Therefore, the protein will spend less time engaged in specific binding with pseudo-sites during its random walk and the average time to locate its true sites will be significantly reduced. In this setting, *R*_*sequence *_can indeed approximate *R*_*frequency *_to a substantial degree. This is a sad coincidence, because it tricks us into assuming that ranking and searching are equivalent problems for the protein. In doing so, we thus disregard any constraints on *R*_*sequence *_imposed by the ranking problem.

By means of a little thought experiment it can be shown that ranking and searching are in fact separate processes operating simultaneously on TF-binding sites. One can easily envision a 22 bp motif for a transcription factor that had 5 totally conserved and 17 equiprobable positions. Such a motif would have an *R*_*sequence *_value of 10 bits, roughly the same amount as *E. coli *CRP. The transcription factor recognizing such a motif would still be able to locate its binding sites with relative high efficiency on an equiprobable background, but it would have no way of gradating its response among them. It would, effectively, have become an on/off switch. Since it is known that this is not the way many transcription factors operate with regard to their sites, one must acknowledge that there are at least two separate processes (affinity ranking and site search) contributing to *R*_*sequence*_. As a matter of fact, it is the way in which these two factors are integrated into *R*_*sequence *_that will determine the degree of equivalence between *R*_*sequence *_and *R*_*frequency*_.

For any given motif size, the values of *R*_*sequence *_at each position yield two obvious limits with regard to the possible range of an affinity ranking function. On the one hand, in a motif with fully conserved positions (maximum motif *R*_*sequence*_) all sites are identical and cannot be differentially regulated, even though they can be located in the genome with the highest efficiency. On the other hand, a motif in which all positions are equiprobable (minimum *R*_*sequence*_) makes it impossible either to distinguish among sites or to discern them against an equiprobable background. Obviously, as in the case of the thought experiment described above, there are many combinations of both situations that also yield minima for ranking range while providing different degrees of search efficiency and *R*_*sequence *_values. Between these extremes, however, there lie a wide scope of combinations providing different *R*_*sequence *_values and affinity ranges. It must be noted, however, that *R*_*sequence *_does not provide direct information on the transcription factor operating range. Instead, this information can be found by examining the distribution of affinity values for each binding site of the prototype group.

**Figure 8:**
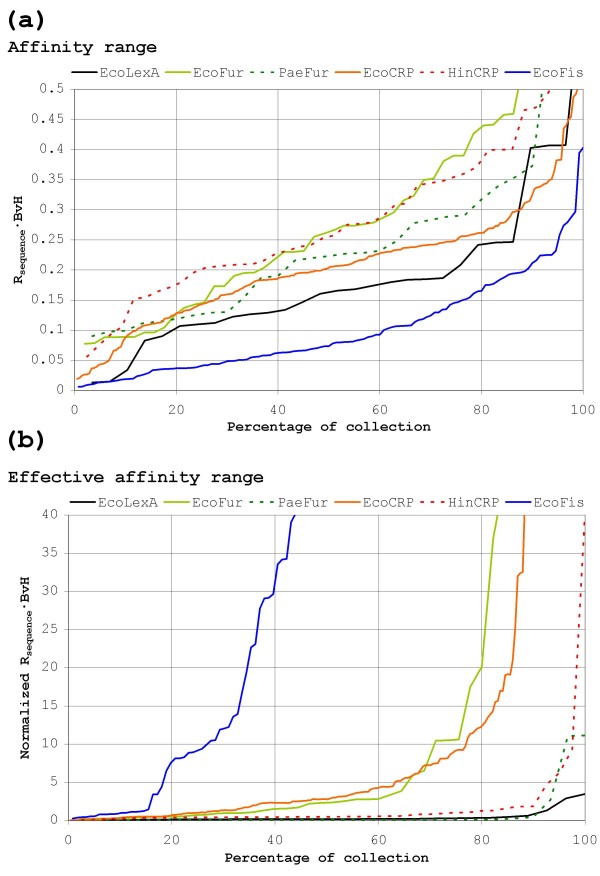
**Standard and effective affinity range for different transcription factors**. (a) Estimation of the affinity range for the different transcription factors analyzed in this work. For each transcription factor, the affinity range is represented as the distribution of affinities for all its experimentally determined binding sites. The affinity of each binding site is estimated using the *R*_*sequence *_· BvH ranking index. (b) Estimation of the effective affinity range. For each transcription factor, the effective affinity range is represented as the distribution of normalized affinities for all its experimentally determined binding sites. Normalized affinities are estimated by normalizing the *R*_*sequence *_· BvH ranking index for each site with the number of false positives required to find that site. For comparison purposes, in both affinity range plots *R*_*sequence *_· BvH values (Y-axis) are normalized to the length of the binding motif for each transcription factor and ranges (X-axis) are shown as the percentage of experimentally determined sites (collection).

Weighted methods were originally developed for ranking binding sites according to their experimental affinity and are thus better suited than non-weighted indices to provide an estimation of affinity range for different transcription factors [27]. Figure 8a shows the affinity ranges based on the *R*_*sequence *_· BvH index for the different transcription factors analyzed in this work. As it has been described previously, *R*_*sequence *_· BvH affinity ranges for transcription factors are highly linear [27]. Therefore affinity ranges can be equated approximately with the slope of their linear correlation, as this captures the dispersion in affinity values. Useful as they are to assess the relative binding affinity of sites, however, affinity plots based on ranking indices like *R*_*sequence *_· BvH do not capture the effective operating range of a protein. This is because they disregard the search problem by focusing exclusively in the prototype group, much in the same manner as the *R*_*frequency *_approach disregards ranking by focusing on search. Clearly, if binding affinity is to be defined meaningfully in a genomic context, it must be defined as the average occupancy of a site by a protein. This suggests that effective binding affinity must be a compound function of both the affinity of the protein for the site (ranking) and its ability to locate it within the genome (search). Such a compound function can be approximated to a certain extent by modulating the ranking index (*R*_*sequence *_· BvH) for each site with the fraction of false positives required to locate it, thus combining information on both the ranking and search processes. The results of this compound function are presented in Figure 8b and they reveal how transcription factors can make use of the search process to alter dramatically the linear shape of their affinity range.

If one assumes an equivalent protein concentration for the different *E. coli *transcription factors shown in Figure 8b, it can be seen that transcription factors targeting motifs with low *R*_*sequence *_values, like Fis, present a strong dispersion in their effective binding affinity, since all but the best sites become rapidly indiscernible from the background. For higher *R*_*sequence *_values, transcription factors can exploit their potential affinity range in different manners, aiming at reaching a balance among site conservation (*R*_*sequence*_), the desired effective affinity range and a viable concentration of transcription factor. Based on the results shown in Figure 8, it can be argued that transcription factors covering a large number of sites, like CRP, sacrifice part of their linear affinity range in order to effectively cover the vast majority of their sites without incurring in a large cost in conservation (*R*_*sequence*_) and protein concentration. On the other hand, transcription factors for more specific responses like Fur, which target a lower number of sites, can maintain higher *R*_*sequence *_values and higher linear ranges. This would allow such transcription factors to operate strongly on a number of sites at the same time as they maintain a much looser control on the rest of the regulon. A biological rationale for this mode of operation can be the necessity to strongly activate/repress several genes important for the specific response while relaying relaxed regulation to less specific genes.

Without prior knowledge of the specific functional requirements for a given transcription factor it is difficult to predict the evolutionary pathway it will follow to meet the equilibrium between *R*_*sequence*_, protein concentration and its effective affinity range. The SOS response repressor LexA, however, poses an interesting case example since a part of its functional requirements is well known. LexA targets around 30 palindromic binding sites in *E. coli *[53] and its binding motif has an *R*_*sequence *_value of 20.27 bits. This has long defied interpretation by the standard information theory approach because *R*_*frequency *_calculations indicate that the LexA binding motif ought to contain 17.39 bits (*R*_*frequency*_). Instead, the observed *R*_*sequence *_suggests that LexA is over-specified to the point of targeting efficiently as few as 4 sites in the *E. coli *genome. Schneider and Stormo suggested that specific binding of other proteins to T7 promoters might account for extreme over-specification in these sites [54], but no such cross-interaction has ever been described for *E. coli *LexA-binding sites. Most probably, the reason for the over-specification of LexA lies in its regulation of the *sulA *gene, encoding a cell division inhibitor that leads to lethal *lexA*^- ^mutant phenotypes, and several DNA damage-inducible error-prone polymerases and DNA helicases that can substantially hamper viability if unregulated [55-58]. As it has been postulated previously, the negative effects of these genes require that they be under very tight repression in normal circumstances [48,56,58,59]. Figure 8b shows that LexA enforces efficient repression of key genes by using a relatively high protein number (1300 molecules per cell) and an unexpected amount of site conservation (*R*_*sequence*_). This allows LexA to operate effectively in its original linear range, as the search process contributes little to the effective affinity range. By maintaining a high ratio (~1/6) with the concentration of inducer (RecA), the system is able to guarantee a fast response time, which is also known to be a requirement of the SOS response [60,61].

**Figure 9:**
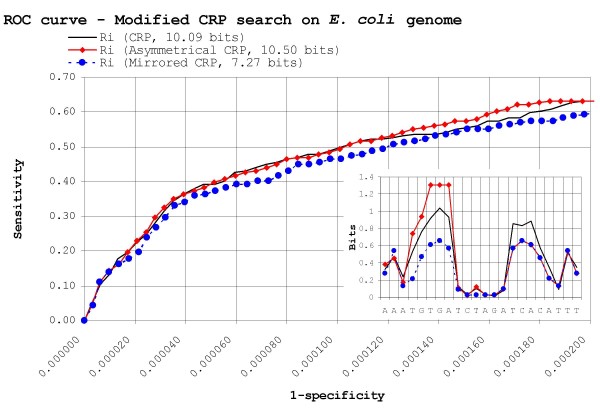
**Search efficiency in *E. coli *with "weakened" CRP sites**. Mean ROC curves for the *R*_*i *_search method trying to locate CRP binding sites on the *E. coli *genome, using the original, asymmetric and mirrored collections of CRP. The plot is scaled to encompass a 1/10 true to false positive ratio for CRP (2100 false positives). The *R*_*sequence *_profile of the original, asymmetrical and mirrored CRP motifs is shown in the inset.

*R*_*sequence *_is by definition a measure of positional information content. This has been interpreted previously by different authors as being either primarily a measure of affinity range [27] or an estimate of search performance [13]. Following the line of reasoning outlined above, however, *R*_*sequence *_provides an averaged measure of the informational requirements for both the search and ranking processes. This suggests that, to some degree, part of *R*_*sequence *_may be devoted to one or the other process. Transcription factors targeting palindromic motifs offer a good benchmark to test this hypothesis. If one assumes a dimer search pathway [62], specificity against the background is obtained mainly by dimer binding, suggesting that approximately the same amount of information should be present in both half-sequences. Nonetheless, many palindromic motifs in *E. coli*, like CRP, exhibit a slightly asymmetrical shape, with one half-sequence more conserved than the other. By reversing known sites on the basis of half-site conservation, it is possible to accentuate this effect, leading to heavily asymmetrical motifs (Figure 9 inset).

Here we assessed the efficiency of the *R*_*i *_search method operating on a collection of weakened CRP sites. The weakened collection (*R*_*sequence *_= 7.27 bits) was derived from the highly asymmetrical CRP motif by substituting the strong half-sequence with a mirror copy of the weak half-sequence for each site (Figure 9 inset). Despite a 31% reduction in information content on an already underspecified motif and the arbitrary introduction of artificial symmetry, the search results of the mirrored CRP collection are quite close (5.5% difference) to those obtained using both the original and asymmetric CRP collections (Figure 9). The fact that similar results are obtained when the same mirroring procedure is applied to other palindromic motifs, like Fnr (data not shown), leads us to suggest that the excess information observed in the strong dyad of asymmetrical palindromic profiles may be used primarily for binding affinity, while search operates mainly on the remaining symmetrical information.

### Reassessing binding site evolution

Given the number of factors governing the equilibrium among *R*_*sequence*_, protein concentration, binding site number and effective affinity range, it is difficult to accurately assess the theory outlined above in the case of skewed genomes. Nonetheless, certain broad predictions can still be made for the evolution of transcription factors trapped in a genome drifting towards skew. As discussed above, based on the equality between *R*_*frequency *_and *R*_*sequence*_, *RE *predicts that anti-skew motifs on a skewed genome are prone to lose some positional information because the search problem is overtly simplified in the skewed genome. As the *P. aeruginosa *Fur case illustrates, however, this does not seem to be the case. In fact, the evidence suggests that, for the transcription factor to fulfill an equivalent function in the skewed genome, its anti-skew motif must retain or even increase its positional information content. This is due to the fact that affinity ranking must now operate in a background in which the search process does not contribute significantly to the effective affinity range (Figure 8b). Transcription factors may adapt partly to this situation by lowering their number of copies in the cell (and thus increasing the relevance of the search process in determining effective affinity), but it is difficult to see how they might shed away positional information, as this would only limit further their operational range.

Transcription factors targeting pro-skew sites face the opposite problem. In this case, the search problem becomes a fundamental limiting factor and high *R*_*sequence *_values are required in order to distinguish sites from the genomic background. Nonetheless, the minimum *R*_*sequence *_value required for efficient location of sites does not guarantee per se a desirable effective range for affinity. Thus, additional information content may still be required to provide an effective affinity range. In particular, one must take into account that, due to the pro-skew composition of sites, any increase in the linear affinity range will result in a very large increase in effective affinity range, as search requirements will very rapidly disrupt the original affinity scope. This suggests that a considerable amount of additional information will be required to maintain an adequate effective affinity range. Although they do not constitute solid proof, the search results for Fur shown in Figure 3 certainly support this hypothesis. These results suggest that 14 bits of information in a 33% GC-rich genome ought to be enough to provide a search efficiency roughly similar to that of CRP in *E. coli*. Nevertheless, *H. influenzae *CRP displays 17.83 bits, indicating that additional information is being used to provide it with an adequate operating range.

From a broader perspective, the fact that *R*_*sequence *_values do not decrease for *P. aeruginosa *Fur and *H. influenzae *CRP is in agreement with a peculiarity of *R*_*sequence *_that has been puzzling researchers for decades. As outlined in the introduction, in skewed genomes *R*_*sequence *_decreases without regard to the direction of the motif skew. The reason is that the background genomic entropy (*H*_*before*_) decreases, making less information available for encoding recognition. Schneider *et al*. argued that by going against the skew a transcription factor might exploit search efficiency and benefit from the skew [13], but in terms of information theory this would be akin to a free lunch proposition: motifs could become more informative in a less informative setting. As we have shown above, however, a skew in genome composition introduces a net reduction in information that influences both the search and ranking problems to different extents. Thus, search might be facilitated by the genomic skew, but at the cost of hampering the effective affinity range. In order to compensate for the overall loss in information content and maintain comparable functionality, transcription factors in skewed genomes are forced to increase or at the least maintain the positional information content of their motifs.

In contrast to the conventional viewpoint, anti-skew sites trapped in a genome drifting towards skew would benefit from moving towards the skew instead of remaining against it. Clearly, if *P. aeruginosa *Fur moved towards the skew, the search problem would gain relevance, yielding a larger effective affinity range for the same positional information. In addition, it can be argued that positional information would be less expensive to maintain for a motif more attuned to the genomic skew. Indeed, *P. aeruginosa *Fur seems to be drifting towards the skew, but its drift appears to be remarkably slow. A possible explanation for this fact is that migration towards the skew implies a co-evolutionary process between a transcription factor and its binding sites that may not be easy to attain without temporal loss of functionality. Given this constraint, maintenance or increase of positional information content against the skew may be a much simpler pathway and thus act as a powerful attractor in the evolutionary landscape faced by transcription factors trapped in genomes drifting towards skew.

### Reassessing binding site search

In the light of the arguments expounded above, it seems apparent that straightforward heuristic improvements and ad-hoc modifications of information theory methods are ill-suited to cope with the inherent complexity of the interactions between transcription factor binding sites and the genomes they sit in. The poor results of *RE*-derived methods in skewed genomes certainly support this idea. Arguably, more complex methods can be used to model the background genomic sequence more accurately, as it is routinely done in motif discovery tools using Markov models [63-65]. This would allow implementing more reliable corrections to improve search in skewed genomes. However, it must be borne in mind that functional affinity range requirements on site conservation may still degrade performance even if accurate background models are used. This is because any background correction becomes effectively a weighting factor in the analysis of putative binding sites. As we have shown here, weighting increases the chances of random false positives by making methods focus on fewer positions. Therefore, any excess weighting due to affinity range requirements on *R*_*sequence *_will tend to increase false positive rates in spite of accurate background corrections. In the light of this, non-weighted methods based on *R*_*sequence *_(*R*_*i*_, *HI*) seem on average the best choice in the general problem of site search because they make the least assumptions (Table 1). The avoidance of prior assumptions is also a characteristic of several machine-learning paradigms, like Artificial Neural Networks (ANN) or Hidden Markov Models (HMM). Due to their iterative training nature, these methods are ideally suited to detect and incorporate into their internal model complex deviations in the genomic background [66,67]. Therefore, they have the potential to match and even outperform information theory methods for site search. Moreover, these methods can also use and infer different types of information (e.g. curvature) encoded within the sequence, as well as existing interdependences between motif positions [67-69]. Still, method standardization and broad applicability remain a thorny issue for these computing paradigms, and a substantial effort in this direction is required before they can be successfully applied to the binding site search problem.

**Table 1:** Summary of relative method performances.

		Random background		Genomic background		
**Method**	**Type**	**Equiprobable**	**Skewed**	**Equiprobable**	**Skewed**	**Reference**

*R*_*i*_	NW	++++	++	++++	++++	[23]
*I*_*seq*_	NW	++++	++++	++++	+++	[25]
*R*_*sequence *_· *BvH* *R'*_*sequence*_	W	++	+	++	+++	[27,28]
*RE · BvH* *RE'*	W	++	+++	++	++	This work
*FitomHI*	W	++	+	++	++	This work

Summary of relative method performance on the search problem against different backgrounds. Increasing numbers of + signs symbolize higher accuracy. *W *stands for weighted and *NW *for non-weighted method.

## Conclusion

The results presented above have several important implications for the understanding of binding site search, information and evolution. On the search problem, we conclude that non-weighted *R*_*sequence*_-based methods should be used preferentially, as they contain fewer assumptions and are thus less prone to misfire on real biological data. Conversely, weighted *R*_*sequence*_-based methods seem to be better indicated to affinity rank sites. Relative entropy and similar heuristic corrections for skew composition should be avoided, since they are based on the misguided hypothesis that search and differential regulation are equivalent problems for the protein. In contrast, we propose that information content as defined by *R*_*sequence *_is a compound measure that incorporates requirements from the search and regulation processes. This revised paradigm suggests that binding sites will tend to drift towards the genomic skew, not against it, and increase their conservation to circumvent the global loss of information content in skewed genomes.

## Methods

### Sequences and collections of binding sites

Complete genome sequences for *E. coli *str. K-12 substr. MG1655 [NC_000913], *P. aeruginosa *PAO1 [NC_002516], *H. influenzae *Rd KW20 [NC_000907], *Colwellia psychrerythraea *34H [NC_003910], *Salinibacter ruber *DSM 13855 [NC_007677], *Thiobacillus denitrificans *ATCC 25259 [NC_007404], *Enterococcus faecalis *V583 [NC_004668], *Anaplasma marginale *str. St. Maries [NC_004842] and *Nitrosococcus oceani *ATCC 19707 [NC_007484] were downloaded from the Entrez database at NCBI .

Collections of binding sites (Table S2, Additional file 1) for *E. coli *Fis, CRP and Fur sites, and for *P. aeruginosa *Fur sites were downloaded from the Prodoric database [70]. The collection of *E. coli *LexA binding sites was obtained from [53]. *H. influenzae *CRP binding sites were provided by Rosie Redfield [71].

### Computer programs

Searches for binding sites using the different methods described herein were conducted entirely with Fitom, a program to locate binding sites in genomic sequences [72]. Fitom allows different modes of action, in which the user can chose on a variety of search methods, threshold adjustments, report styles and background entropy calculations. For the purposes of this work, all searches were carried using the computed background entropy of the full genome sequence [23] and no small sample correction [13] in the estimation of *R*_*sequence *_and *RE*.

Random backgrounds with different skews were generated with RandSeq, a simple program written in C++ to generate random sequences based on a naïve Bernoulli model of mononucleotide frequencies. To simulate search processes on random backgrounds, binding sites from experimentally validated collections (Table S2, Additional file 1) were inserted at known positions in the randomly generated sequences. Frequencies for 20-mers in real and artificial genomes were computed with NmerFreq. Executable programs, user manuals and source code are available for download at .

### ROC curves

All reported ROC curves correspond to simulated search processes, either on real genomic sequence or on artificially generated sequence. In a simulated search process, a collection of experimentally validated binding sites is provided to Fitom and the program scans both strands of the target sequence. Experimentally validated binding sites present in the target sequence are considered positives. All other sites in the target sequence are considered negatives. For a given threshold *θ*, sensitivity is computed as the ratio between true positives (positives reported as positives by Fitom according to *θ*) and positives. Likewise, specificity is computed as the ratio between true negatives (negatives reported as negatives by Fitom according to *θ*) and negatives. ROC curves of simulated search processes on artificially generated sequence show the mean and standard deviation of three independent experiments (on three different randomly generated sequences).

## Authors' contributions

IE conceived and designed the study, carried out all programming and data collection and drafted the manuscript. MCO verified data and results, helped to draft the manuscript and participated in the design of the study. Both authors read and approved the final manuscript.

## Supplementary Material

Additional file 1 **Table S2.** Table showing information logos for different TF-binding motifs. Click here for file
