# Supplementary material for: A reexamination of information theory-based methods for DNA-binding site identification
Source: BMC Bioinformatics. 2009 Feb 11;10:57. doi: 10.1186/1471-2105-10-57 (PMC2680408; doi:10.1186/1471-2105-10-57)
Supplement: Additional file 1 — Table S2. Table showing information logos for different TF-binding motifs. [file 1471-2105-10-57-S1.doc]

Table S1 – Information logos for different TF-binding motifs

| TF | Genome | Logo | Sites | *R_sequence_ (bits)* | Source |
| --- | --- | --- | --- | --- | --- |
| CRP | *Eco* | 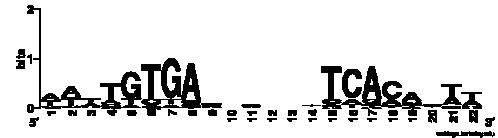 | 210 | 10.09 | [73] |
|  | *Hin* | 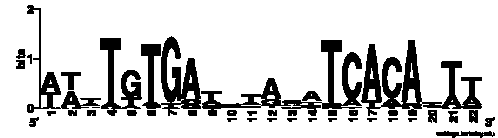 | 45 | 17.83 | [71] |
| Fur | *Eco* | 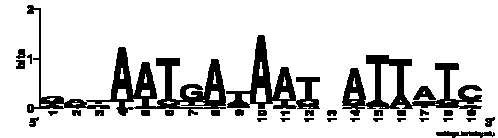 | 51 | 14.31 | [73] |
|  | *Pae* | 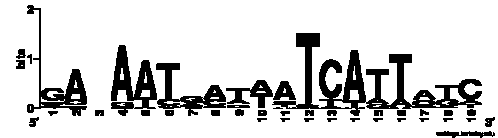 | 32 | 13.69 | [73] |
| LexA | *Eco* | 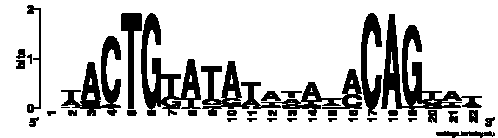 | 27 | 21.33 | [53] |
| Fis | *Eco* | 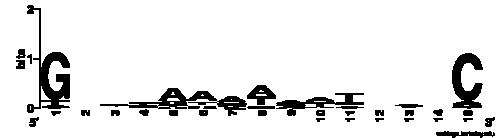 | 116 | 5.16 | [73] |

**Information content profiles for the different TF-binding motif analyzed in this work. Sequence logos [74] were generated using the WebLogo server at** <http://weblogo.berkeley.edu/> **[75]. *R_sequence_* values were computed with *Fitom*, using the real genome background entropy. Abbreviations: Eco – *E. coli*, Hin – *H. influenzae*, Pae – *P. aeruginosa*.**
